# Spatio-Temporal Distribution of Negative Emotions in New York City After a Natural Disaster as Seen in Social Media

**DOI:** 10.3390/ijerph15102275

**Published:** 2018-10-17

**Authors:** Oliver Gruebner, Sarah R. Lowe, Martin Sykora, Ketan Shankardass, SV Subramanian, Sandro Galea

**Affiliations:** 1 Department of Geography, Humboldt-Universität zu Berlin, Berlin 10099, Germany; 2 Epidemiology, Biostatistics, and Prevention Institute (EBPI), University of Zurich, 8001 Zurich, Switzerland; 3 Department of Psychology, Montclair State University, Montclair, NJ 07043, USA; lowes@mail.montclair.edu; 4 Centre for Information Management (CIM), School of Business and Economics (SBE), Loughborough University, Loughborough LE11 3TU, UK; M.D.Sykora@lboro.ac.uk; 5 Department of Health Sciences, Wilfrid Laurier University, Waterloo, ON M5B 1W8, Canada; ketan.shankardass@gmail.com; 6 Department of Social and Behavioral Sciences, Harvard T.H. Chan School of Public Health, Boston, MA 02115, USA; svsubram@hsph.harvard.edu; 7 School of Public Health, Boston University, Boston, MA 02118, USA; sgalea@bu.edu

**Keywords:** advanced sentiment analysis, digital epidemiology, geographic information system, geo-social media, hotspots, post-disaster mental health, psychogeography, spatial epidemiology, spatial regimes regression, Twitter data

## Abstract

Disasters have substantial consequences for population mental health. We used Twitter to (1) extract negative emotions indicating discomfort in New York City (NYC) before, during, and after Superstorm Sandy in 2012. We further aimed to (2) identify whether pre- or peri-disaster discomfort were associated with peri- or post-disaster discomfort, respectively, and to (3) assess geographic variation in discomfort across NYC census tracts over time. Our sample consisted of 1,018,140 geo-located tweets that were analyzed with an advanced sentiment analysis called ”Extracting the Meaning Of Terse Information in a Visualization of Emotion” (EMOTIVE). We calculated discomfort rates for 2137 NYC census tracts, applied spatial regimes regression to find associations of discomfort, and used Moran’s I for spatial cluster detection across NYC boroughs over time. We found increased discomfort, that is, bundled negative emotions after the storm as compared to during the storm. Furthermore, pre- and peri-disaster discomfort was positively associated with post-disaster discomfort; however, this association was different across boroughs, with significant associations only in Manhattan, the Bronx, and Queens. In addition, rates were most prominently spatially clustered in Staten Island lasting pre- to post-disaster. This is the first study that determined significant associations of negative emotional responses found in social media posts over space and time in the context of a natural disaster, which may guide us in identifying those areas and populations mostly in need for care.

## 1. Introduction

Large-scale natural disasters are observed worldwide [1,2,3] and can have substantial consequences for population mental health [4,5,6,7]. Although research to date has documented a great deal of mental health resilience in the aftermath of disasters, elevated rates of mental health consequences, including depression and post-traumatic stress, have been observed [7,8,9,10]. One of the key risk factors for adverse post-disaster mental health outcomes is experiencing overwhelming emotions in the context of disaster, which may be caused by the event itself, such as by exposure to trauma (i.e., actual or threatened death, serious injury, or sexual violence) [9]. These emotions may also be influenced by non-traumatic *stressors* either in the short or long term, such as loss of financial property, personal belongings, and/or one’s home [4,7,9,10,11]. For example, emotions may convey information about how people evaluate what is happening and how they cope with it when they experience stress [11]. Sadness is usually related to experiencing an irreversible loss, and fright related to perception of immediate, concrete, and overwhelming physical danger [11]. Many studies have shown such negative emotional responses during a disaster being predictive of mental health problems in the aftermath of disaster [4], which may reflect the impact of disaster in terms of experienced stress. It is, however, important to differentiate “normal responses to abnormal events” from a lack of mental health resilience because the majority of people who report negative emotional reactions during a disaster do not develop mental health problems in the aftermath [7,10]. Nevertheless, we might expect the spatial and temporal distribution of emotional responses to follow the location and timing of disaster impacts, with implications for the burden of related adverse mental health problems.

The majority of studies on disaster mental health to date have relied solely on post-disaster data. Post-disaster studies are prone to possible recall bias, with the consequence that we are potentially missing important information on the time before the event. Some notable studies to date have included pre-event data [12,13,14]. Although these studies have brought a much better understanding of changes in mental health from pre- to post-disaster, research on this topic would further benefit from information that is gathered both prior to and during the event. Such information could, for example, demonstrate multiple dimensions of emotional reactions over time in the context of a disaster that could increase the precision of data relevant to mental health responses. Specifically, investigating multiple specific emotions, as defined by Ekman [15], such as fear, anger or disgust, or a combination thereof, could inform about the quality and strength of emotions as they are experienced during the disaster and could inform about risk for possible post-disaster mental health outcomes [16,17,18,19,20,21,22,23].

Research to date has provided little insight into geographic variability in where emotions are excessively expressed during or in the aftermath of large-scale disasters. Geographic concentrations of emotional expression would be indicative of some form of stress that is jointly experienced in specific populations within areas at specific times. Information on the concentration of specific emotional responses may exhibit people’s alertness, and geographically mapping those places could reveal the spatial extent of areas in which possible issues arise guiding early intervention to minimize adverse mental health consequences. Better knowledge about the location and timing of specific emotional concentrations could provide important information about how to more efficiently roll out emergency public health interventions in the aftermath of disasters and other mass traumatic events. 

Social media data, such as from Twitter, may help to fill these gaps, as they are largely and publicly available, have been successfully applied to disaster research, and provide pre-, peri-, and post-disaster information [23,24,25]. For example, specific emotions, such as fear or sadness, can be detected from Twitter streams through advanced sentiment analysis [26,27] and can be related to periods before, during, and after disasters. Furthermore, such data provide the opportunity for the geographic assessment of single posts. Hence, ecological momentary assessment of emotional reactions—that is, ongoing evaluation of in-the-moment experiences—becomes possible [26]. For example, in a study on emotional responses in the greater New York City area during Superstorm Sandy, Gruebner et al. [21] used Twitter data and found specific negative emotions clustered over space and time. However, this previous study used a relatively narrow time frame during the disaster.

We set out to explore the spatial distribution of *discomfort* in the population before, during, and after Superstorm Sandy that formed over the Caribbean on 22 October, hit the New York City (NYC) area on 29 October, and disappeared on 2 November 2012 [28]. The storm caused 43 deaths and about US$ 19 billion in damages within NYC alone making it the most costly and destructive disaster to impact public housing in the history of this city [29]. Specifically, we aimed to (1) identify a composite of negative emotions (i.e., discomfort) as expressed by the population that posted geo-located Twitter tweets in NYC over three time periods, each of approximately two weeks, representative of the pre-disaster period, the peri-disaster period, and the post-disaster period. We further aimed to (2) assess whether pre- or peri-disaster discomfort were associated with peri- or post-disaster discomfort, and to (3) investigate whether there was geographic variation in discomfort risk across NYC boroughs over time.

## 2. Materials and Methods 

We used Twitter for this study because it is a publicly available resource that can be widely used for research purposes. Furthermore, tweets were geo-referenced facilitating spatial analysis informing our research questions. In addition, Twitter has already been used in other studies in that context [21,30,31,32].

About 45 million social media users that were mostly under the age of 50 in the United States were monthly active on Twitter by the end of 2012 [33,34,35]. Approximately three percent of them potentially used geolocation services producing geo-located Twitter data [36]. We used geo-located Twitter data published within NYC for the time frame of 10 October to 18 November 2012. Our sample was composed of 1,018,140 tweets that were suitable for our analysis, i.e., had information about geographic locations from where the tweets had been issued and were in English (see Table 1). We used tweets that were geo-located within those NYC census tracts that shared a border in order to facilitate spatial analyses at the census tract level. Thereby, 399,089 tweets were within the pre-disaster period (8 October to 21 October), 235,423 tweets were within the peri-disaster period (22 October to 4 November), and 383,628 tweets were within the post-disaster period (5 November to 18 November). Our level of analysis was the census tract (*N* = 2137). The dataset was available from the Harvard Center for Geographic Analysis Geo-tweet Archive (CGA) [37], the institution that collected the data. For transparency in research, the Tweet IDs used in this study can be made available for interested researchers, according to Twitter’s sharing policy. Harvard provides a rehydration app to facilitate conversions of TweetIDs back to full tweets [38].

We first analyzed the raw data with the advanced sentiment detection program “Extracting the Meaning Of Terse Information in a Visualization of Emotion” (EMOTIVE) [25,39]. While standard sentiment analysis tools only separate the mood as identified from social media texts into negative, positive, or neutral, EMOTIVE is able to detect basic emotions as defined by Ekman [40], such as *anger, disgust, fear, happiness, sadness, surprise*, and also *shame* and *confusion,* thereby preserving the original tweet texts and timestamps. We combined six of these emotions that are typically considered as negative emotions, i.e., *anger, confusion, disgust, fear, sadness*, and *shame* into one single emotion which we named *discomfort* to maximize statistical power. We coded each tweet dichotomously for the presence (case = 1) or absence (no case = 0) of discomfort. We then separated the dataset into three sets of two weeks each, representative of the pre-disaster period (8 October to 21 October), the peri-disaster period (22 October to 4 November), and the post-disaster period (5 November to 18 November). We then noted all discomfort cases at the census tract level and calculated smoothed spatial empirical Bayes (SEB) rates using the percentage of tweets that were indicative of discomfort out of all tweets (tweet population) for each census tract during each time period. This method was used to adjust for heterogeneity of variances of the rates [41] that evolved due to varying population sizes in the total tweets across NYC’s census tracts. Empirical Bayes rates were calculated in GeoDa (Center for Spatial Data Science, Chicago, IL, USA) [41]. In addition, we checked whether SEB rates were significantly different across pre-, peri-, and post-disaster with paired t-tests in R (The R Foundation, Vienna, Austria) [42].

Second, we assessed associations between pre- and peri-disaster discomfort and between pre- as well as peri- and post-disaster discomfort rates at the census tract level initially with Ordinary Least Squares (OLS) regression models. Because OLS residuals exhibited spatial autocorrelation indicated by a Lagrange multiplier test, with the lag model performing well over the spatial error model, we chose a spatial lag model (Spatial Two Stage Least Squares regression (S2SLS)) as suggested in Anselin and Rey [43]. This model explicitly included a spatial lag variable on the right-hand side of the regression equation to account for the spatial structure found in the data stemming from non-independence of discomfort SEB rates in neighboring census tracts. 

Third, we investigated spatial clusters of above average SEB (discomfort) across the census tracts at each time period by applying spatial autocorrelation analyses (Global and local Moran’s I) in GeoDa [41]. We then ran a series of spatial regimes regression models (S2SLS) to further account for structural instability [43], that is, geographic variation in the associations between pre- and peri-disaster discomfort and post-disaster discomfort across NYC boroughs, i.e., the regimes. Furthermore, a Chow test [44] was applied for regime diagnostics. Regression analysis was applied in GeoDaSpace (Center for Spatial Data Science, Chicago, IL, USA) [45].

## 3. Results

### 3.1. Rates of Discomfort in Twitter Data

**Figure 1:**
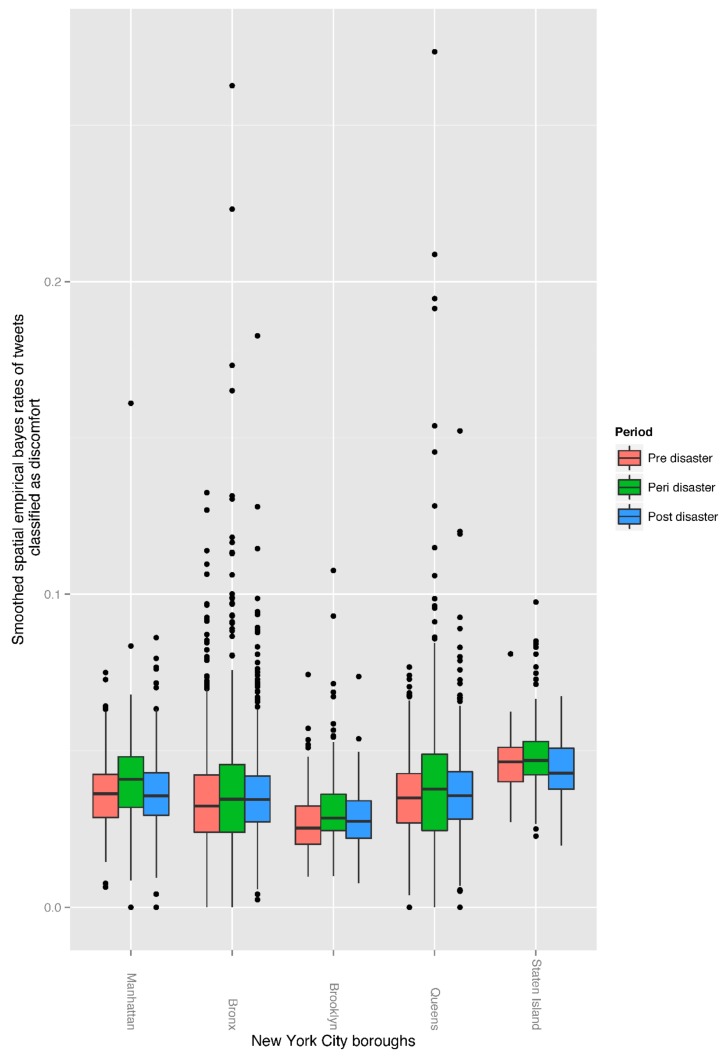
Spatial Empirical Bayes smoothed rates of tweets classified as discomfort for each NYC borough across time periods.

We extracted and combined six negative emotions from the Twitter activity of users in the given area and time frames in a single index that we termed *discomfort*. We identified 2649 cases (i.e., tweets) (0.66%) indicative of discomfort pre-disaster, 1641 cases (0.70%) peri-disaster, and 2845 cases (0.74%) post-disaster. We noted that compared to pre-disaster, overall discomfort rates were significantly different peri-disaster with *t*(2136) = −6.65, *p* <0.001 and a mean of difference of −0.003. Post-disaster rates were also significantly different from pre-disaster rates *t*(2136) = −3.06, *p* = 0.002 (mean of difference = −0.001) and from peri-disaster rates *t*(2136) = 4.76, *p* < 0.001 (mean of difference −0.002). In addition, we noted that median rates of discomfort were different across boroughs and time periods, with the highest rates across all time periods in Staten Island as compared to the other boroughs, and Brooklyn having the lowest rates across all time periods compared to the other boroughs (Figure 1).

### 3.2. Associations of Discomfort Over Time

In multivariable regression models, we found a significant association between pre-disaster discomfort and post-disaster discomfort (Beta = 0.11, *p* < 0.001) as well as between peri-disaster discomfort and post-disaster discomfort (Beta = 0.10, *p* < 0.001) (see Table 2). Pre-disaster discomfort and peri-disaster discomfort were, however, not significantly associated. Additionally, both peri- (Beta = 0.68, *p* < 0.001) and post-disaster discomfort (Beta = 0.61, *p* < 0.001) were spatially interdependent across neighboring census tracts, that is, peri- and post-disaster levels of discomfort in one census tract were significantly associated with those in adjacent census tracts.

### 3.3. Spatial Variation in Discomfort Risk

**Figure 2:**
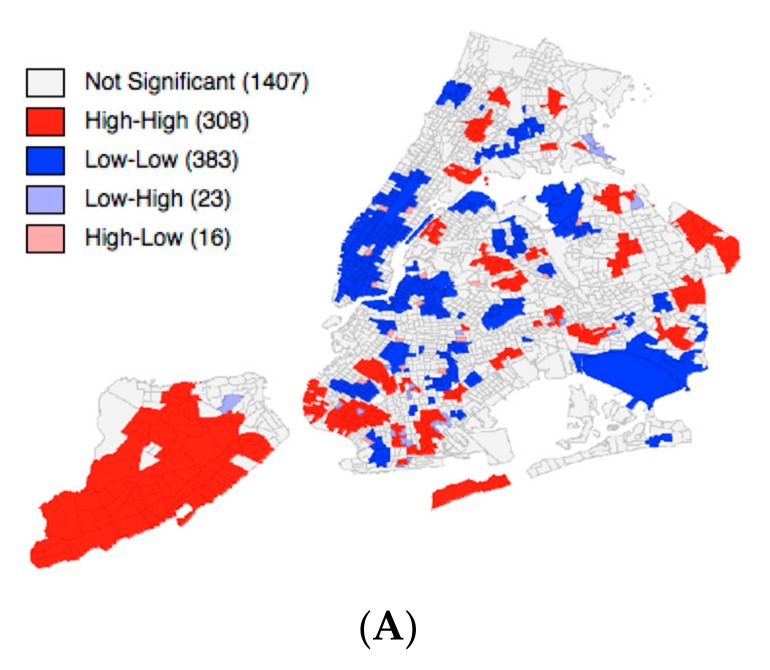

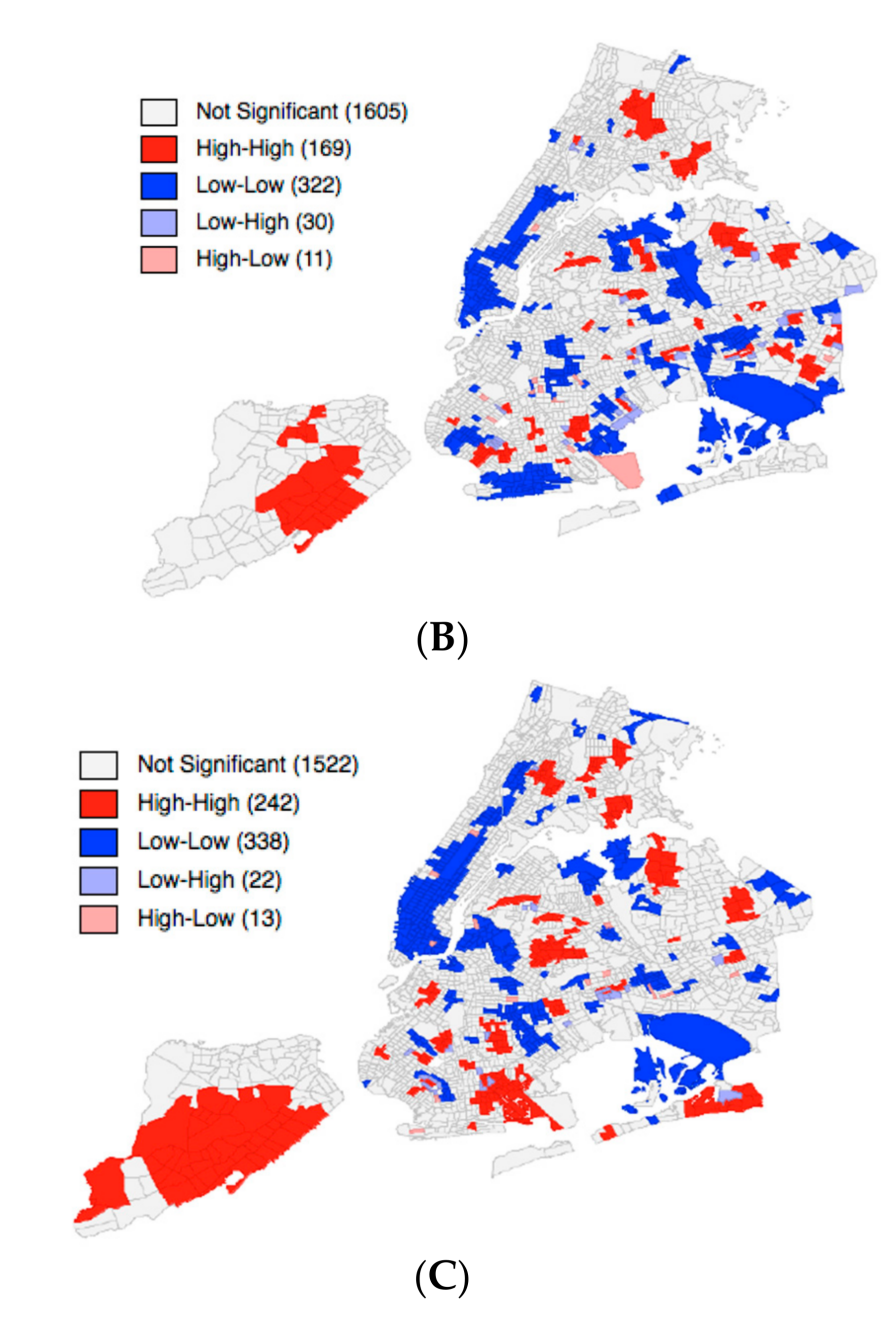
Local clusters of above average discomfort rates (shaded in red) in NYC geo-located Twitter tweets for the three periods before (**A**), during (**B**) and after (**C**) Superstorm Sandy, indicating that high rates were found next to other high rates (High-High). Rates were smoothed with the spatial empirical Bayes smoother prior to the cluster analysis. Notably, the statistic also calculates below average rates (low rates next to other low rates) and outliers (high rates next to low rates and vice versa) that are not considered in this study.

We found significant spatial clusters of above average rates of discomfort in all time frames, i.e., pre-, peri-, and post-disaster (Figure 2). The most prominent cluster of discomfort in terms of size was located in Staten Island and was persistent at all time periods, albeit with a varying number of census tracts that were included in the cluster. Although Chow tests did not reveal significant structural instability across the boroughs with spatial regimes regression, we noted that pre-disaster discomfort was significantly and positively associated with post-disaster discomfort in Manhattan (Beta = 0.18, *p* < 0.01), the Bronx (Beta = 0.22, *p* < 0.001), and Queens (Beta = 0.09, *p* < 0.01) (Table 3). Furthermore, peri-disaster discomfort was also significantly and positively associated with post-disaster discomfort in Manhattan (Beta = 0.13, *p* < 0.01) and Queens (Beta = 0.10, *p* < 0.05). In addition, peri- (Beta = 0.91, *p* < 0.001) and post-disaster discomfort (Beta = 0.64, *p* < 0.001) remained spatially interdependent across neighboring census tracts.

## 4. Discussion

We found negative emotional reactions that we combined in one variable, which we called discomfort. Overall, discomfort rates were significantly different after the disaster as compared to before or during the pre-disaster period across boroughs. Further, we showed that pre- and peri-disaster discomfort were significantly associated with post-disaster discomfort rates. Moreover, discomfort rates were spatially clustered across NYC census tracts and associations of discomfort rates over time were different across boroughs with significant associations only in Manhattan, the Bronx, and Queens. 

The Twitter activity of users can exhibit perceived and real risk as experienced by users [21,31]. Our results show that there were higher rates of discomfort expressed in Tweets during the post-disaster period as compared to during the peri-disaster period, likely corresponding to the time period in which residents had to deal with the greatest stressors (e.g., losses) after the storm. Staten Island had the highest median rates of discomfort among all boroughs and across all time frames, which was consistent with what one might expect based on the variability of Sandy’s impact across NYC. Staten Island was one of the boroughs that was hit the hardest by Superstorm Sandy due to a combination of the storm’s arrival and high tide leading to high levels of inundations and crucial service outage after the storm [46]. All the above might explain why we see highest rates of discomfort in Staten Island.

Taking into account all census tracts in a global regression model, we found that higher volumes of specific negative emotions at pre- or during the disaster were associated with negative emotions also after the disaster. From studies that compared mental health symptoms from before with after the disaster we know that pre-disaster conditions are one of the strongest predictors for post-disaster conditions [12,13,14]. This may also be true for emotional responses as expressed on Twitter and other social media platforms.

We further investigated geographic concentrations in discomfort rates across NYC regions (i.e., census tracts) within and across the boroughs and found significant spatial clustering of above average discomfort rates in all boroughs except Manhattan, with biggest clusters across census tracts in Staten Island. Spatial clusters may occur due to spatial dependence of rates across census tracts. Our analysis did not investigate the possible causes of small-area variation in discomfort over time; however, we have at least three possible explanations for spatial dependence causing the clusters. First, there were environmental issues, such as flooding and houses affected by the storm with some of them being totally destroyed [47]. Further, reported power shortages, falling trees, or strong winds spanning across census tracts may have affected a wider population in several neighboring census tracts simultaneously. This might have been particularly true across several neighboring census tracts in Staten Island, where considerable flooding and destroyed houses have been reported by the Federal Emergency and Management Agency (FEMA) [47,48]. While people collectively experienced these environmental issues, they may have processed feelings in the affected areas leading to these spatial patterns of negative emotions. There has also been more localized environmental damage, such as floods and affected houses in parts of other boroughs, for example in the Queens areas spanning from Averne to the Far Rockaway, where we found clusters, too [47,48].

Second, daily interaction of, for example, residents of one census tract with facilities of other census tracts (e.g., coffee shop, work place, shelter, drop-off location for receiving aid and goods) while tweeting might have produced spatial patterns of emotions spanning across several adjacent census tracts.

Third, people may have perceived environmental issues differently depending on their socio-demographic characteristics or socio-ecological context in which they live, for example with people living in better structural quality of housing worrying less about strong winds. Socio-demographic characteristics or socio-ecological contexts vary substantially across NYC boroughs (with some neighborhoods being highly segregated across census tracts), which may have further contributed to the clusters. Future studies should include neighborhood level socio-demographic and socio-ecologic variables to further understand these patterns.

We also found that clusters of above average discomfort rates in several census tracts were persistent over time from peri- to post-disaster. The most prominent clusters at all time periods spanning across the largest number of census tracts were also located in Staten Island. These clusters and those elsewhere may exhibit areas of increased risk for mental health needs due to, e.g., increased environmental issues, mobility (or displacement) of tweeters, different perceptions among tweeters, or a combination of the three.

In addition, we investigated the spatial variability of associations of discomfort rates over time. We found that pre-disaster discomfort was significantly associated with post-disaster discomfort only in Manhattan, the Bronx, and Queens, while peri-disaster discomfort was significantly associated with post-disaster discomfort only in Manhattan and Queens. We may expect other factors being more important predictors for post-disaster discomfort in the other boroughs, such as neighborhood-level socio-ecological factors (e.g., socio-economic status, physical exposure to the storm). With regard to the boroughs in which we found significant positive associations of discomfort rates across the time periods, our results may indicate that the associations were place dependent with socio-ecological factors specific to local census tracts and boroughs. Our results may also indicate that those areas with higher discomfort rates during the storm—presumably due to issues such as service outfalls or strong winds—may be more likely to exhibit discomfort also after the storm, when neighborhoods were flooded or houses seriously affected by the storm. Since early emotional reactions predict post-disaster mental health problems [4], this should be investigated in more depth in future studies.

Our study had several limitations. First, we used geo-located English tweets from Twitter. Future studies may also include non-geo referenced data including further languages or data sources. Second, due to likely service shortages during and in the immediate aftermath of the storm, the three time frames under investigation had different number of tweets with the lowest number of tweets during the disaster, which should be kept in mind when interpreting the findings. For example, it is possible that Twitter users were unable to charge their phones, that they were trying to conserve battery life, or that they were restricted in their cell phone usage due to interrupted cell phone services, hence limiting the activity of Twitter users during that time. Third, we only used tweets from NYC census tracts that shared a border with another NYC census tract to facilitate spatial analysis. This excluded tweets that were published in census tracts not directly connected to other census tracts (e.g., Ellis Island) or that were posted while travelling on water. Fourth, since tweeters were assumingly using mobile devices while tweeting, it is possible that tweets were not always sent from the same location but rather while on the daily commute, at home, at work, or in a shelter before or during the storm. After the storm, some of the Twitter population may have been displaced and would be tweeting from other locations as before or during the storm. Finally, we have worked at the ecological level, that is, census tracts in which tweets have been posted rather than at the individual Twitter user level. Use of individual Twitter identifiers would permit the application and testing of more traditional paradigms of stress response variation during a trauma as predicting post-trauma distress.

## 5. Conclusions

This is the first study that identified emotions representative of discomfort in social media along with their concentration in space and time. Discomfort, i.e., negative emotional responses including amongst others fear, anger, and sadness, were concentrated in some neighborhoods across NYC and were persistent over time, most prominently so in Staten Island. These concentrations may provide knowledge about areas and communities with mental health need. 

High discomfort rates pre-disaster were associated with high rates during and after the disaster. The association of pre-, peri-, and post-disaster discomfort over time was place dependent, suggesting different socio-ecological factors responsible for discomfort across the five boroughs.

Since early emotional reactions may predict longer-term mental health needs, this approach could further assist in the long-term allocation of services. EMOTIVE is currently being extended to also evaluate stress responses that in conjunction with a spatially explicit approach, as it has been applied here may help to estimate the development of symptoms indicative of depression and PTSD [49,50]. Given that social media use has dramatically increased worldwide since 2012, these data provide enormous potential for mental health research to study e.g., the functional relationship between socio-ecological factors and mental health. For example, working at the individual social media user level might inform about individual people’s exposure in addition to weather-related damage and flooding, such as loss of resources and restricted access to services in real time. In addition, this level of analysis could also capture other community-level risk factors such as geographic variation in pre-existing mental health conditions or social support. In countries with limited formal surveillance infrastructure, the approach may also have potential for the identification of areas and populations in need for care.

## Figures and Tables

**Table 1:** Descriptive figures for aggregated Tweets at the census tract level over the entire study period in New York City from 10 October–18 November 2012. For example, the range of tweet population (or individual emotions) indicates the minimum and maximum number of all tweets, or of tweets with a specific emotion, found in census tracts. Note that discomfort is a combination of the negative emotions anger, confusion, disgust, fear, sadness, and shame. Tweets were coded as discomfort when they were indicative of any of these emotions at the individual level. Therefore, the numbers do not sum up at the census tract level when single emotions are compared to discomfort at that level.

Variable	Range	1st/3rd Quintile	Median	Mean	Sum
Tweet population	1–6507	24/149	59	158.8	1,018,140
Discomfort	0–158	0/5	2	5.03	32,254
Anger	0–119	0/1	0	0.77	4918
Confusion	0–8	0/0	0	0.25	1620
Disgust	0–45	0/1	0	1.11	7126
Fear	0–34	0/1	0	0.59	3841
Sadness	0–77	0/3	1	2.24	14,333
Shame	0–23	0/0	0	0.17	1079

**Table 2:** Spatial regression results. Model 1 is a spatial lag regression model.

Variable	Peri-Disaster Discomfort		Post-Disaster Discomfort	
	Coef.	S.E.	Coef.	S.E.
**Model 1: New York City**				
NYC Intercept	0.00	0.01	0.01 **	0.00
Pre-disaster discomfort	0.03	0.04	0.11 ***	0.02
Peri-disaster discomfort	/		0.10 ***	0.01
Spatial lag of peri-disaster discomfort	0.68 ***	0.18	/	
Spatial lag of post-disaster discomfort	/		0.61 ***	0.07
**Model diagnostic**				
Pseudo R-squared	0.33		0.47	
Spatial Pseudo R-squared	0.03		0.13	
Anselin-Kelejian Test	1.99		2.67	

Coef. = Coefficient estimate; S.E. = Standard Error; Significance level: *** <0.001, ** <0.01.

**Table 3:** Spatial regression results. Model 2 is a spatial lag regression model with regimes, i.e., the boroughs.

Variable	Peri-Disaster Discomfort		Post-Disaster Discomfort	
	Coef.	S.E.	Coef.	S.E.
**Model 2: New York City boroughs**				
Manhattan Intercept	0.00	0.01	0.00	0.00
Pre-disaster discomfort	0.09	0.12	0.18 **	0.06
Peri-disaster discomfort	/		0.13 **	0.04
Bronx Intercept	0.00	0.01	0.00	0.00
Pre-disaster discomfort	0.07	0.07	0.22 ***	0.07
Peri-disaster discomfort	/		0.05	0.04
Brooklyn Intercept	0.00	0.01	0.01	0.00
Pre-disaster discomfort	0.01	0.06	0.07.	0.04
Peri-disaster discomfort	/		0.10	0.06
Queens Intercept	0.00	0.01	0.01	0.00
Pre-disaster discomfort	0.03	0.08	0.09 **	0.04
Peri-disaster discomfort	/		0.10 *	0.05
Staten Island Intercept	0.01	0.01	0.01 *	0.01
Pre-disaster discomfort	−0.03	0.09	0.07	0.09
Peri-disaster discomfort	/		0.04	0.04
Global spatial lag of peri-disaster discomfort	0.91 ***	0.25	/	
Global spatial lag of post-disaster discomfort	/		0.64 ***	0.14
**Model diagnostic**				
Pseudo R-squared	0.33		0.47	
Spatial Pseudo R-squared	0.03		0.14	
Chow test for intercept	1.84		9.09	
Chow test for pre-disaster discomfort	1.97		6.99	
Chow test for peri-disaster discomfort	/		4.01	
Global Chow test	2.36		18.06	
Anselin-Kelejian Test	1.93		1.04	

Coef. = Coefficient estimate, S.E. = Standard Error, Significance level: *** <0.001, ** <0.01, * <0.1.
